# Imaging Review of Pelvic Ring Fractures and Its Complications in High-Energy Trauma

**DOI:** 10.3390/diagnostics12020384

**Published:** 2022-02-02

**Authors:** Edoardo Leone, Andrea Garipoli, Umberto Ripani, Riccardo Maria Lanzetti, Marco Spoliti, Domenico Creta, Carolina Giannace, Antonio Galluzzo, Margherita Trinci, Michele Galluzzo

**Affiliations:** 1 Department of Emergency Radiology, Azienda Ospedaliera San Camillo-Forlanini, 00152 Rome, Italy; margherita.trinci@libero.it (M.T.); mgalluzzo@scamilloforlanini.rm.it (M.G.); 2 Department of Radiology, Università Campus Bio-Medico di Roma, 00128 Rome, Italy; gar.andreag@gmail.com; 3 Department of Emergency and Major Trauma, Division of Anaesthesia, Analgesia and Intensive Care and Pain Therapy, Ospedali Riuniti di Ancona, 60162 Ancona, Italy; umberto.ripani@ospedaliriuniti.marche.it; 4 Orthopaedics and Traumatology Unit, Department of Emergency and Acceptance, Azienda Ospedaliera San Camillo-Forlanini, 00152 Rome, Italy; riccardolanzetti@gmail.com (R.M.L.); marcodoc@me.com (M.S.); 5 Physical Medicine and Rehabilitation Service, Casa di Cura Privata Madre Fortunata Toniolo, 40141 Bologna, Italy; info@domenicocreta.it; 6 UOC SISP, ASL Roma 1, 00135 Rome, Italy; carolinagiannace@gmail.com; 7 Department of Radiology, Università Degli Studi di Firenze, 50121 Florence, Italy; antoniogalluzzo17@gmail.com

**Keywords:** pelvic ring fracture, high-energy trauma, computed tomography, intrapelvic bleeding, genitourinary injury

## Abstract

Pelvic ring fractures are common in high-energy blunt trauma, especially in traffic accidents. These types of injuries have a high rate of morbidity and mortality, due to the common instability of the fractures, and the associated intrapelvic vascular and visceral complications. Computed tomography (CT) is the gold standard technique in the evaluation of pelvic trauma because it can quickly and accurately identify pelvic ring fractures, intrapelvic active bleeding, and lesions of other body systems. To properly guide the multidisciplinary management of the polytrauma patient, a classification criterion is mandatory. In this review, we decided to focus on the Young and Burgess classification, because it combines the mechanism and the stability of the fractures, helping to accurately identify injuries and related complications.

## 1. Introduction

Fractures of the pelvic ring are common in high-energy blunt trauma, mostly in road traffic accidents. In polytrauma patients, these fractures, especially when unstable, cause intrapelvic vascular and visceral injuries, and are associated with a high mortality rate. Injuries of the other body systems (brain, chest, upper abdomen) are often associated with pelvic ring fractures.

Starting from the description of the relevant anatomy and biomechanics of the bony pelvis, this review aims to describe the main classifications of pelvic ring fractures (with a particular focus on the Young and Burgess one) and the most common intrapelvic vascular and visceral related complications.

## 2. Epidemiology of Pelvic Trauma

Pelvic fractures are widely considered to be one of the most complex and fatal lesions, accounting for 2–8% of all skeletal injuries [1,2].

The incidence of pelvic trauma is increasing due to the high number of traffic collisions involving pedestrians, motorcyclists, and cyclists [3]. Other causes of pelvic trauma are work and sports accidents.

In the case of high-energy blunt trauma, the average age of pelvic fractures is between 30 and 50 years. In high-energy blunt trauma, pelvic ring fractures are rarely isolated, but they are often associated with injuries of other organs: brain, lung, spleen, liver, kidney, long bones, and thoracic aorta [4,5]. The overall mortality of this kind of trauma is 5–16%, and it is related to hemodynamic instability, sepsis, and multiorgan failure [6].

For low-energy trauma, the age is higher (about 65 years), and mortality is lower. The lesion is usually due to an osteopenic/osteoporotic condition without significant complications or associated lesions [7,8].

## 3. Relevant Anatomy and Biomechanics of the Bony Pelvis

The bony pelvis has a ring morphology, consisting of the connection of the sacrum with the two innominate bones. Each innominate bone is formed by the ilium, the ischium, and the pubis.

The anterior part of the pelvic ring is composed of the superior and inferior pubic rami and the pubic symphysis. The pubic symphysis is a non-synovial joint, composed of a fibrocartilaginous disc between the two pubic surfaces [9]. The pubic symphysis allows very little movement, and it has the biomechanical function of stabilizing the anterior pelvis and preventing the collapse of the pelvis when standing [10].

The posterior part of the pelvic ring is composed of the sacrum, the iliac wings, and the sacroiliac joint. The sacroiliac joint is covered by fibrocartilage on the iliac surface and by hyaline cartilage on the sacral surface [11]. The posterior part of the pelvic ring serves to transmit the load from the spine to the lower limbs [11].

It is important to remember that the osteoarticular structures of the pelvic ring have no inherent stability. For example, the pubic symphysis provides only 15% of pelvic stability [12]. Indeed, pelvic stability depends mainly (ratio of 8:2) on the pelvic ligaments of the posterior tension band: anterior and posterior sacroiliac ligaments, iliolumbar ligament, sacrospinous ligament, and sacrotuberous ligament. The iliolumbar, anterior sacroiliac, and sacrospinous ligaments guarantee rotational stability, thanks to their horizontal course [13,14]. The posterior sacroiliac and sacrotuberous ligaments ensure vertical and anteroposterior stability, due to their vertical course [13,14].

In consideration of its ring morphology, a lesion in one point will always correspond to a second lesion on the opposite side. There are only two exceptions to this rule: avulsion fractures in young people and insufficiency fractures in the elderly, where even mild trauma can result in rupture of the pelvic ring at one point [15].

## 4. Classification of Pelvic Ring Fractures

In the evaluation of pelvic trauma, the first step is to distinguish pelvic ring fractures from acetabular fractures and avulsion fractures.

Once the pelvic ring fracture is recognized, it is essential to assess its stability. Fractures and dislocations of the posterior part of the pelvic ring, and in particular the degree of involvement of the posterior tension band, affect the stability of the pelvic ring [14,16].

Many classifications of pelvic ring fractures have been proposed with the aim of guiding treatment and determining prognosis.

### 4.1. Pennal Classification

Pennal was the first to propose a classification based on the mechanism of fracture [17]. This classification system recognizes three mechanisms of the direction of the vector force, in pelvic ring fractures:

- Anterior–posterior compression.
- Lateral compression.
- Vertical shear.

### 4.2. Tile Classification

In 1988, Tile proposed an alphanumeric classification system, which gave great importance to the stability mechanism [18]. By dividing the pelvic ring into an anterior and a posterior arch (anterior and posterior to the acetabulum, respectively), he classified the pelvic ring fractures into three types, with different degrees of stability. Each type has multiple subtypes, which depend on the bone component involved [13].

- Type A fractures are stable because the posterior arch of the pelvic ring is intact.
- Type B fractures have rotational instability, but they are vertically stable, due to an incomplete disruption of the posterior arch structures.
- Type C fractures are vertically and rotationally unstable, and they are characterized by a complete disruption of the anterior and posterior arches.

### 4.3. Young and Burgess Classification

The Young and Burgess classification combines the mechanism and the stability of the fractures [19]; it is also a very useful guide in the search for associated intrapelvic vascular and visceral complications, and in the management of polytrauma patient (Table 1).

The first step in using this system is the identification of the main direction of the force vector: lateral compression (50–70% of cases), anterior–posterior compression (20–30% of cases), vertical shear (14% of cases), and combined mechanism [20]. These categories have different grades of severity, based on the magnitude of the force, the pelvic structures involved, and the vascular and visceral pelvic complications [21].

#### 4.3.1. Lateral Compression

Injuries in which the force vector causes a lateral compression are the most common cause of pelvic ring fracture, and they are often seen in pedestrians hit by a car [20]. These kinds of forces cause an internal rotation of the hemipelvis and a reduction of the pelvic volume. Lateral compression injuries typically cause bladder rupture and sacral nerves lesions, while pelvic hemorrhages are less common.

**Figure 1:**
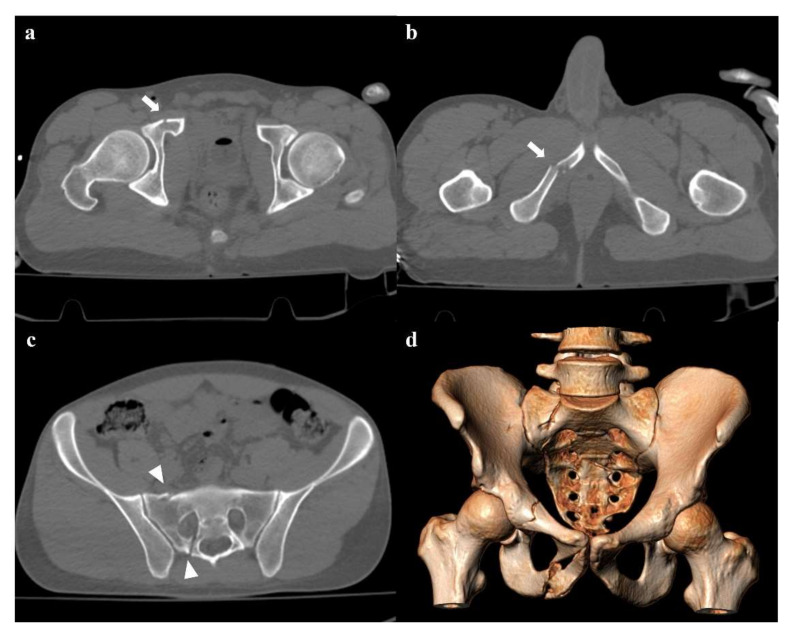
Lateral compression fracture, type 1. Axial computed tomography (CT) images show a fracture of the right-side superior and inferior pubic rami (arrows in (**a**,**b**)), and ipsilateral fracture of the sacrum (arrowheads in (**c**)); the sacral fracture involves the neuroforamina (zone II). Three-dimensional volume-rendering CT reconstruction in anteroposterior (AP) view confirms the type of fracture (**d**).

**Figure 2:**
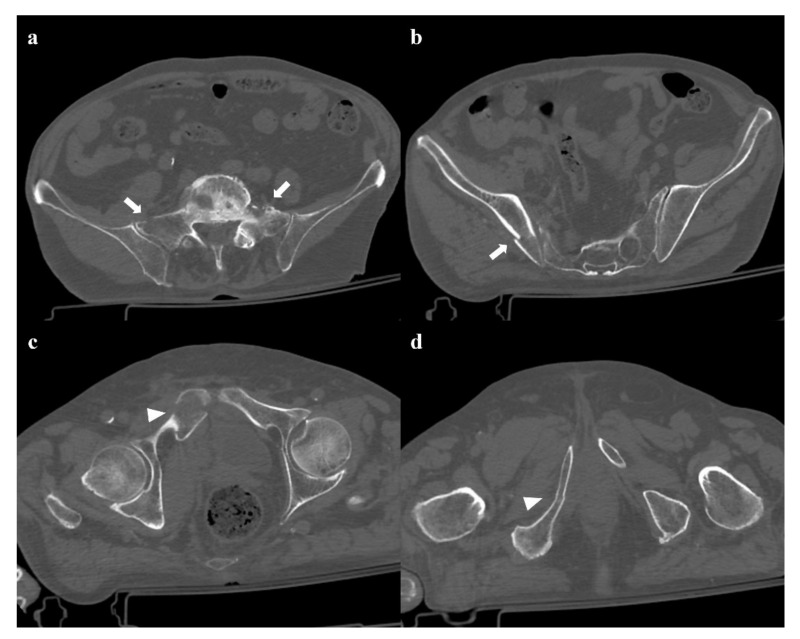
Lateral compression fracture, type 2. Axial CT images show a fracture of both the sacral wings (arrows in (**a**)), without the involvement of the neuroforamina (zone I). There is also fracture of the iliac wing on the right side (arrow in (**b**)), and ipsilateral fracture of the superior and inferior pubic branches (arrowheads in (**c**,**d**)).

**Figure 3:**
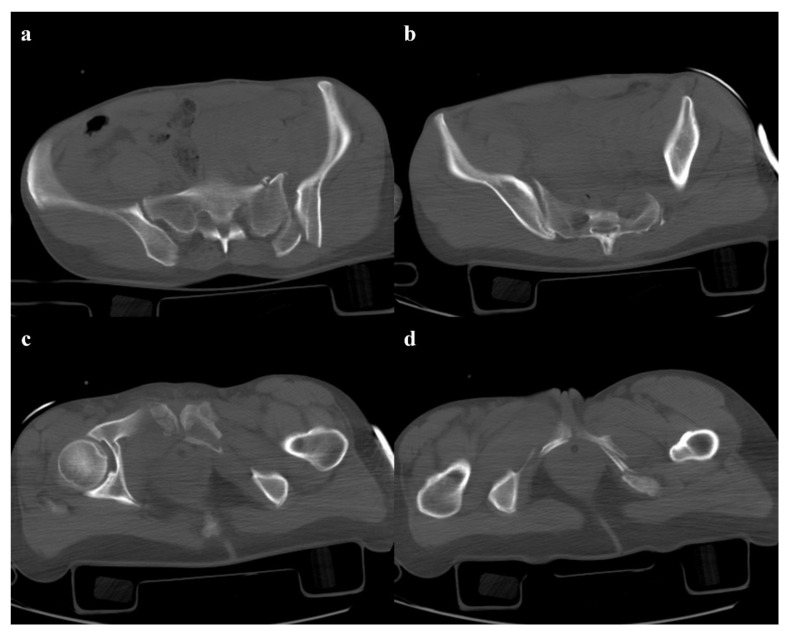
Lateral compression fracture, type 3. Axial CT images show the fracture of the iliac and sacral wings on the left side (**a**), anterior widening of the right sacroiliac joint (**b**), and fracture of bilateral pubic branches (**c**,**d**).

Lateral compression injuries have three degrees of severity:

- Grade 1: compression fracture of the sacrum on the side of the impact, and transverse fracture of unilateral or bilateral pubic branches (Figure 1). Lateral compression grade 1 injuries are stable and have a nonoperative management [3].
- Grade 2: grade 1 fractures, iliac wing fracture, posterior sacroiliac joint diastasis (Figure 2). Lateral compression grade 2 injuries have a rotational instability and require a stable internal fixation [3]. In these lesions, an adjunctive temporary external fixation is useful [3].
- Grade 3: the lateral compression force is associated with a contralateral anteroposterior compression force, with an external rotation of the contralateral hemipelvis (“windswept pelvis”). Grade 1 or grade 2 lateral compression injuries are associated with a contralateral sacroiliac joint diastasis (Figure 3). Lateral compression grade 3 injuries have a multidirectional instability and require a stable internal fixation [3]. In these lesions, an adjunctive temporary external fixation is useful [3].

#### 4.3.2. Anterior–Posterior Compression 

The compression force acts with an anterior–posterior vector, generally on the symphysis, causing an external rotation of one or both the hemipelvis and an increase of the pelvic volume [20]. Road traffic accidents typically cause this kind of trauma, which are called “open book” fractures. Pelvic bleeding and hypovolemic shock are possible complications of anterior–posterior compression injuries. For these reasons, “open book” fractures need to be quickly treated with a pelvic wrapping, to reduce the pelvic volume.

Pubic symphysis diastasis is the pathognomonic feature of this type of trauma, with a gap > 1 cm. A widening > 2.5 cm is typically associated with a lesion of the pelvic ligaments of the posterior tension band, causing instability of the pelvis. Pubic symphysis diastasis is sometimes associated with bladder and urethral complications.

**Figure 4:**
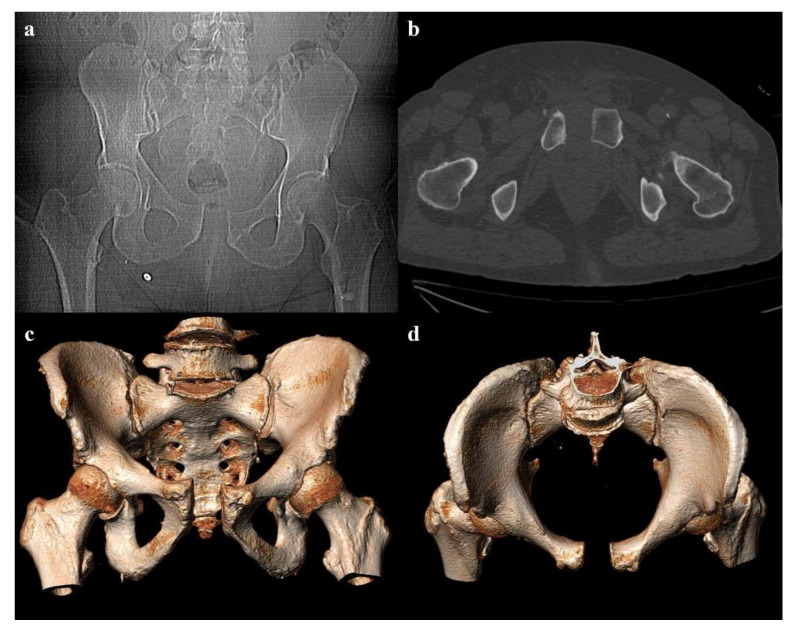
Anterior–posterior compression fracture, type 1. Anteroposterior scout CT view (**a**) and axial CT image (**b**) show a 2.4 cm diastasis of the pubic symphysis. Three-dimensional volume-rendering CT reconstructions in outlet and inlet views (**c**,**d**) well depict the diastasis.

**Figure 5:**
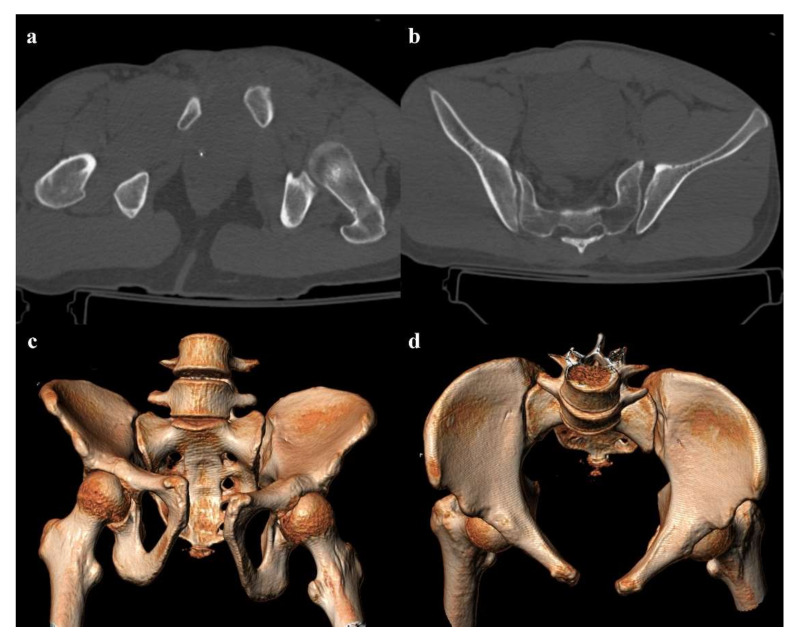
Anterior–posterior compression fracture, type 2. Axial CT images (**a**,**b**) show a pubic symphysis diastasis of 3.2 cm, and an anterior sacroiliac joint diastasis on the left side. Three-dimensional volume-rendering CT reconstructions in outlet and inlet views (**c**,**d**) confirm the findings.

**Figure 6:**
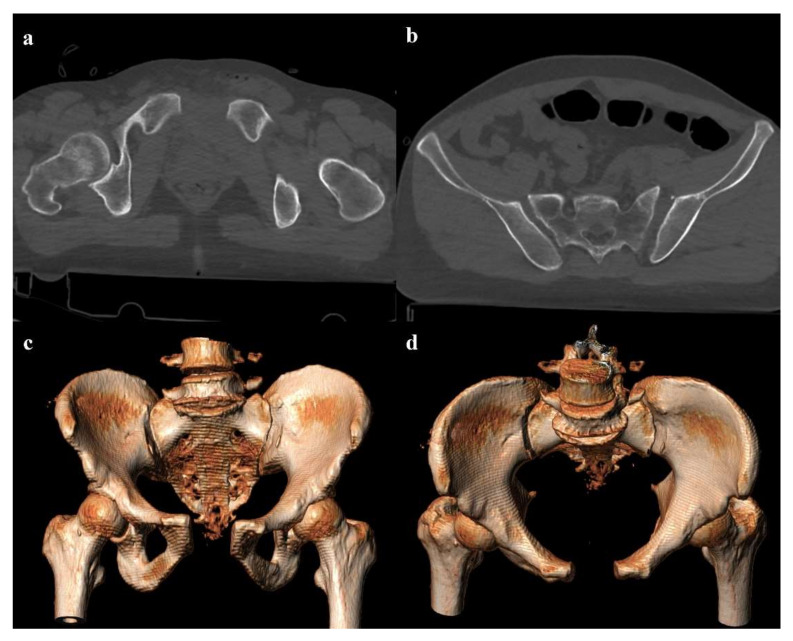
Anterior–posterior compression fracture, type 3. Axial CT images (**a**,**b**) and three-dimensional volume-rendering CT reconstructions in AP and inlet views (**c**,**d**) show a pubic symphysis diastasis of 4.2 cm, anterior and posterior left side sacroiliac joint diastasis, and anterior widening of the right sacroiliac joint.

Anterior–posterior compression injuries have three degrees of severity:

- Grade 1: pubic symphysis diastasis <2.5 cm, possible vertical fracture of the pubic rami (Figure 4). Anterior–posterior compression grade 1 injuries are stable and have a nonoperative management [3].
- Grade 2: pubic symphysis diastasis >2.5 cm, possible vertical fracture of the pubic rami, and anterior sacroiliac joint diastasis (Figure 5). The anterior sacroiliac joint diastasis is due to the rupture of the anterior sacroiliac ligament, sacrospinous ligament, and sacrotuberous ligament. Anterior–posterior compression grade 2 injuries have a rotational instability and require a stable internal fixation [3].
- Grade 3: pubic symphysis diastasis >2.5 cm, possible vertical fracture of the pubic rami, and anterior and posterior sacroiliac joint diastasis (Figure 6). Anterior–posterior compression grade 3 injuries have a multidirectional instability and require a stable internal fixation [3].

#### 4.3.3. Vertical Shear 

**Figure 7:**
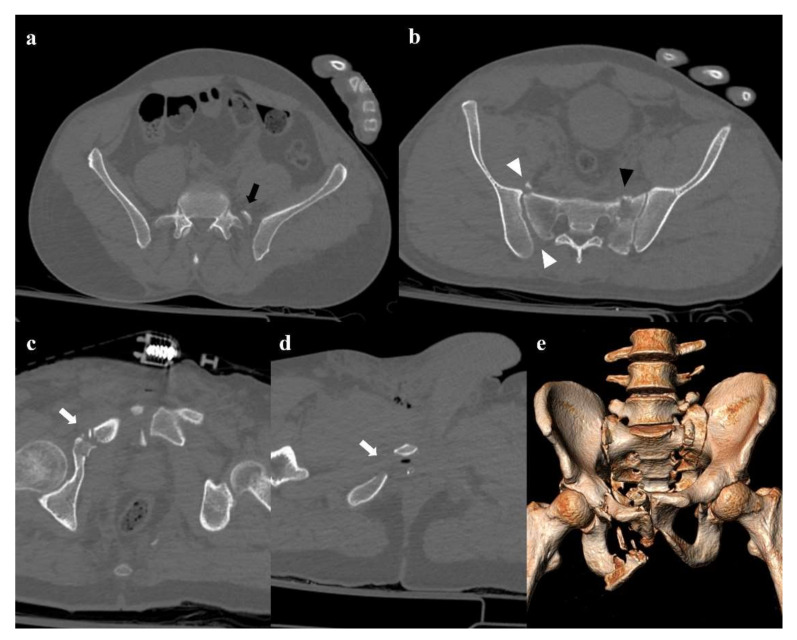
Vertical shear fracture. Axial CT images show avulsion of the left transverse process of the fifth lumbar vertebra (black arrow in (**a**)), fracture of the left side of the sacrum with involvement of the foraminal zone (black arrowhead in (**b**)), and fracture of the right superior and inferior pubic branches (white arrows in (**c**,**d**)). There is also a little fracture of the right wing of the sacrum, and a posterior widening of the right sacroiliac joint (white arrowheads in (**b**)). Three-dimensional volume-rendering CT reconstruction clearly depicts the instability of the pelvic ring, with the fracture of the left transverse process of the fifth lumbar vertebra and the cranial shift of the left hemipelvis (**e**).

A force vector acting in the craniocaudal direction (typically, a fall from a height) can cause a destruction of the posterior elements of the pelvis and a cranial shift of the hemipelvis [13]. Involvement of the posterior arch of the pelvic ring is characterized by the avulsion of the transverse process of the fifth lumbar vertebra on the impact side, which is a sign of iliolumbar ligament rupture (Figure 7). Along the posterior arch, vertical shear injuries can also cause vertical fracture of the sacrum, sacroiliac diastasis, and iliac wing fracture. Anteriorly, this kind of trauma may show a disruption of the pubic symphysis and vertical fracture of the pubic rami. Rotational and vertical instability is the result of vertical shear injuries, that are also associated with vascular, genitourinary, and neurological complications [14]. Vertical shear injuries require a surgical fixation [3].

#### 4.3.4. Combined Mechanism

Rarely, fractures of the pelvic ring are the result of a combined mechanism, especially in very high-energy trauma. The combination of lateral compression and vertical shear is the most common type of combined mechanism injury [14]. Combined mechanism injuries are unstable and require a surgical fixation [3].

### 4.4. Sacral Fractures 

Sacral injuries are part of pelvic ring fractures and are rarely isolated. The fracture is often longitudinal, while it is rarely transverse or combined [22]. One of the most used classifications was proposed by Denis et al. [23]. Denis classification divides the sacrum into three zones, and it is based on the direction, location, and level of fracture with an emphasis on neurologic injury [24].

**Figure 8:**
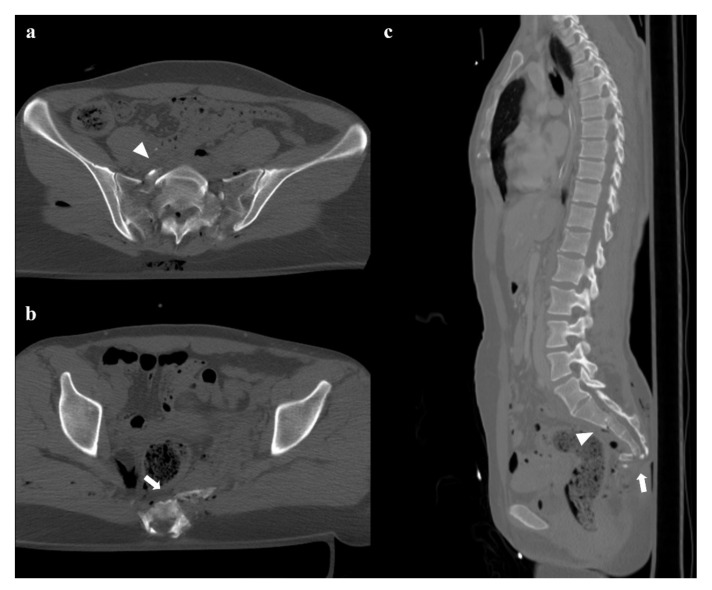
Fractures of the sacrum with involvement of the central canal. Fracture through S2, with anterior angulation of the superior fragment and without dislocation (arrowheads in (**a**,**c**)). Fracture of S4 with complete anterolisthesis of the fragments (arrows in (**b**,**c**)). There are also some bubbles of air in the adjacent soft tissues.

- Zone I: the fracture is located in the sacral wing, lateral to the neuroforamina (Figure 2). In 6% of cases, there is an impingement of L5 or S1 nerve root.
- Zone II: the fracture involves the neuroforamina (Figure 1). In 28% of cases, there is an ipsilateral neurological deficit.
- Zone III: the fracture is medial to the neuroforamina and involves the central canal. A combined fracture in this zone may have different morphology (“H”-shaped, “U”-shaped, “λ”-shaped, “T”-shaped), and can result in a spinopelvic dissociation (Figure 8). Zone III fractures can also be divided into four additional groups: anterior angulation of the fracture without dislocation (type 1), anterior angulation of the fracture with retrolisthesis (type 2), complete anterolisthesis of the fracture fragments (type 3), and comminuted fracture of S1 or S2 (type 4) [22]. In zone III fractures, there is a neurological injury in 56% of cases, with bowel and genitourinary dysfunction [8].

Another useful classification is the AOSpine Sacral Injury Classification System, which combine the morphology of the fracture and the neurological status of the patient with other specific modifiers (soft tissue injury, metabolic bone disease, anterior pelvic ring injury, sacroiliac joint injury) [25]. From a morphological point of view, this classification divides the fractures into three types. Each type has three or four subtypes [25].

- Type A: an injury of lower sacrococcygeal spine, below the level of the sacroiliac joint. These fractures have no impact on the spinopelvic stability.
- Type B: a posterior pelvic fracture, characterized by a unilateral vertical sacral fracture. In these fractures, the posterior pelvic stability may be compromised.
- Type C: spinopelvic fractures, which are unstable.

## 5. Imaging Evaluation

The radiological assessment aims to define the type of fracture, to recognize the degree of instability of the pelvic ring, and to identify possible vascular and visceral complications, to allow the clinician to choose the most suitable treatment [26].

Extended focused assessment with sonography for trauma (e-FAST) plays a central role in the primary management of the polytrauma patient, enabling the evaluation of hemoperitoneum, pneumothorax, pericardial tamponade, and hypovolemic shock [27,28,29].

In the case of pelvic trauma, e-FAST has a low sensitivity in the evaluation of possible retroperitoneal hemorrhage [30,31]. However, it helps to determine potential symphyseal diastasis, showing a high correlation with computed tomography (CT) measurements [32,33].

In hemodynamically unstable patients, plain radiography of the pelvis in the anteroposterior projection can highlight important findings, such as diastasis of the pubic symphysis, fractures of the superior and inferior pubic rami, and avulsion fracture of the fifth lumbar transverse process [34,35].

**Figure 9:**
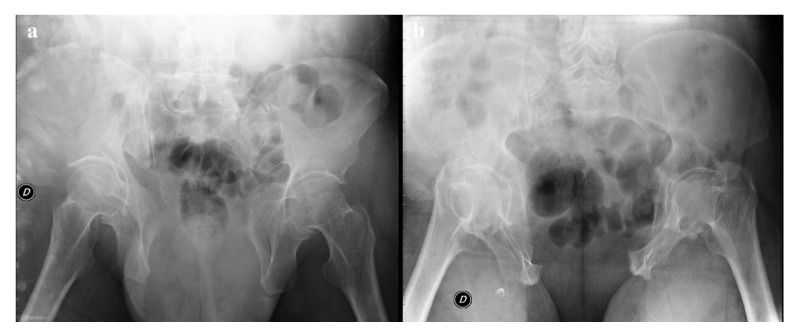
Plain radiography. The outlet view (**a**) and the inlet view (**b**) show a 5.2 cm diastasis of the pubic symphysis and anterior widening of the left-side sacroiliac joint. These findings have to be quickly reported to allow prompt treatment.

Plain radiography is also important in the follow-up of pelvic fractures, by performing anteroposterior and oblique outlet and inlet projections (Figure 9). The outlet view is obtained by directing the inclination of the x-ray beam tilt from the feet at an angle of 35° to the x-ray table, and it allows detection of sacral fractures and craniocaudal dislocations. The inlet view is obtained by directing the inclination of the X-ray beam tilt from the head at an angle of 35° to the X-ray table, and it allows detection of any anteroposterior displacement of the sacroiliac joint or any rotation of a hemipelvis [17,36].

CT is the gold standard technique in the evaluation of high-energy blunt trauma when patient has no hemodynamic abnormalities [34]. In the case of a pelvic involvement, CT can quickly and accurately identify pelvic ring fractures, active bleeding, and bladder rupture [37,38]. Concerning pelvic and sacral fractures, coronal and sagittal multiplanar (MPR) images and three-dimensional volume-rendering reconstructions allow to identify the number, position, and size of bone fragments, and to define the degree of instability [14,39].

## 6. Vascular and Visceral Complications

Pelvic fractures are frequently associated with vascular and visceral complications, such as pelvic bleeding, bladder and urethral lesions, testicular lesions, and peripheral nerve lesions [40]. The risk of vascular and visceral complications is more common in unstable fractures of the pelvic ring [41].

### 6.1. Vascular Complications

Intrapelvic bleeding is an early complication of pelvic fractures, leading to hypovolemic shock and early death in 5–18% of severe trauma [42].

Bleeding has a venous source in 80–90% of cases, due to a lesion of perivesical veins or presacral venous plexus, or a bone fracture [43,44].

In 10–20% of cases, intrapelvic bleeding has an arterial source, from branches of the external or internal iliac arteries [45,46].

**Figure 10:**
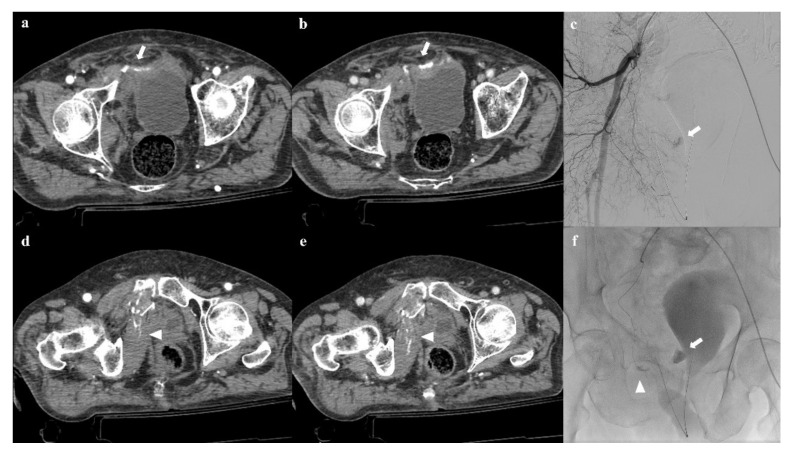
Pelvic ring trauma, characterized by fracture of right superior and inferior pubic rami. Axial CT image in the arterial phase shows active extravasation of contrast medium near the right superior pubic branch (arrow in (**a**)), which increases in the venous phase of the examination (arrow in (**b**)). Similar active extravasation is documented near the internal obturator muscle (arrowheads in (**d**,**e**)). Selective angiography confirmed the two blood extravasation spots, with origin from branches of the right obturator artery (arrows and arrowhead in (**c**,**f**)); both the bleeding spots were optimally embolized.

**Figure 11:**
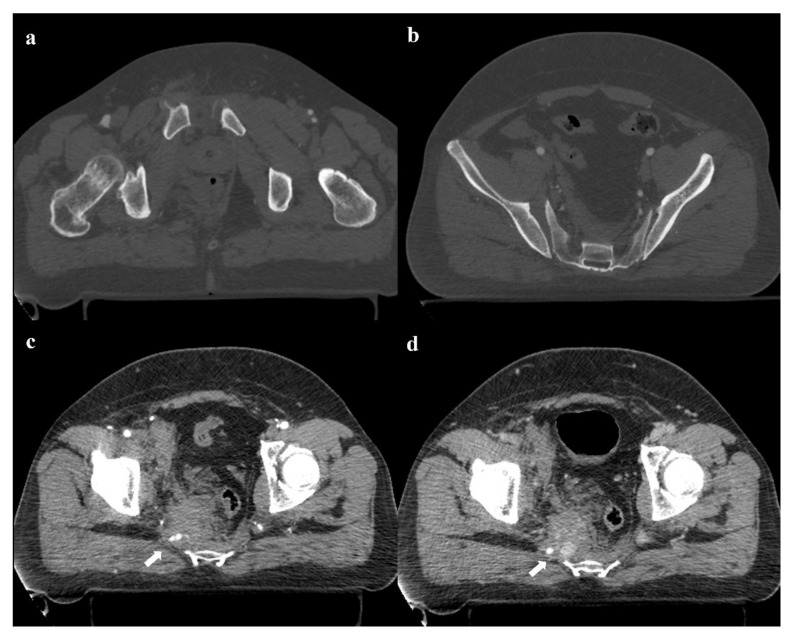
Axial CT images show a pubic symphysis diastasis of 2.9 cm, and an anterior widening of the right sacroiliac joint (**a**,**b**). Active extravasation of contrast medium in the arterial phase of the CT exam is seen near the right side of the fifth sacral vertebra (arrow in (**c**)). The extravasation increases in the next phase of the examination (arrow in (**d**)). This is acute arterial bleeding from a branch of the right inferior gluteal artery.

Active bleeding in the region of the pubic symphysis and pubic rami indicate an injury of the internal pudendal artery, the obturator artery, or inferior epigastric artery (Figure 10). Instead, active bleeding near the iliac wing and the sacroiliac joint indicates an injury of the superior or inferior gluteal artery, iliolumbar artery, or sacral arteries (Figure 11) [47].

Multiphasic CT examination is crucial in identifying active bleeding and in the differentiation between a venous or arterial origin because the management is different.

Venous hemorrhages are often slow-growing and self-limited, and they can be treated conservatively with blood transfusion, pelvic package, or the use of pelvic binders [48].

Arterial hemorrhages require an angiographic embolization or pelvic packing in the case of a hemodynamically unstable patient in absence of the angiographic service [49,50].

Acute arterial bleeding is characterized by active extravasation of contrast medium in the arterial phase of the CT exam, and its attenuation value is like the aorta’s one [51,52]. This hyperattenuating extravascular collection has an irregular shape and tends to increase in the following phases of the examination, allowing the differential diagnosis between active bleeding and vascular malformation, such as pseudoaneurysm and arteriovenous fistula [53].

Pelvic trauma can also be associated with vessel injury, in absence of active bleeding. Other types of traumatic vessel injuries are arterial thrombosis, arterial dissection, intramural hematoma, pseudoaneurysm, and arteriovenous fistula [54,55].

### 6.2. Bladder and Urethral Complications

The bladder and urethra are injured in 3.4% and 1% of cases of pelvic trauma [56]. They are more common when diastasis of the pubic symphysis and displacement of fragments of fractured pubic branches occurred [3,57].

There are four types of bladder injuries: contusion, intraperitoneal rupture, extraperitoneal rupture, and combined rupture [58]. Extraperitoneal rupture is the most common bladder injury (80–90% of cases) in association with anterior pelvic ring fractures and is treated conservatively [59]. On the other end, intraperitoneal rupture is rare and has a surgical treatment [60].

**Figure 12:**
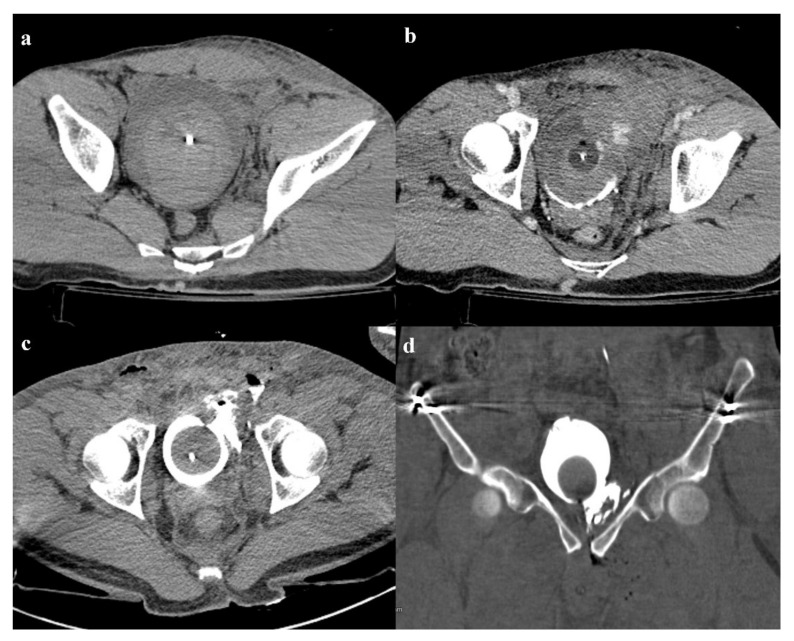
Patient of the Figure 5, with an anterior–posterior compression fracture. There is a large hematoma inside the bladder (**a**); contrast medium administration reveals active bleeding in the venous phase of the examination, on the left wall of the bladder (**b**). Axial and coronal CT cystography images show a leak of contrast medium in the perivesical space, suggestive of extraperitoneal bladder rupture (**c**,**d**).

In the evaluation of bladder lesions, the most accepted diagnostic tool is CT cystography with the retrograde introduction of 300 mL of diluted water-soluble iodinated contrast medium [61,62]. The retrograde approach allows the detection of any bladder rupture, through contrast extravasation in the perivesical space or in the peritoneal space (Figure 12).

**Figure 13:**
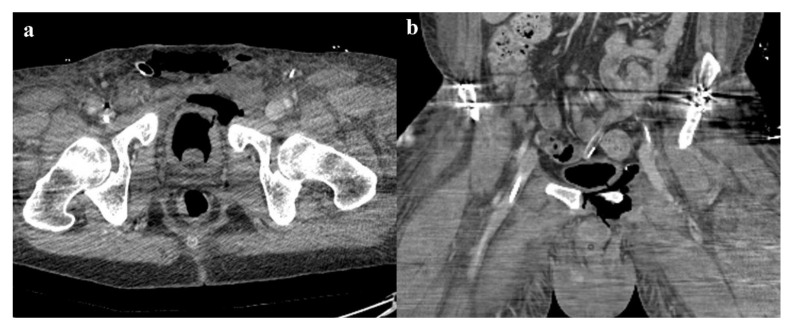
Patient of the Figure 6, with an extraperitoneal bladder rupture. The retrograde introduction of air shows a leak at the level of the anterior wall (**a**,**b**).

Pneumo-CT cystography with the retrograde introduction of 150–300 mL of air is an alternative in the diagnostic management of bladder injuries, and it seems to be more confident in the detection of anterior wall rupture (Figure 13) [63].

Urethral injuries are more common in men and are typically localized in the prostatic and membranous portions [59,64].

Urethrography and CT cystography allow the detection of urethral rupture. Other indirect signs of urethral trauma are hematoma of the ischiocavernosus and internal obturator muscles, and fluid in the fat tissue near the urogenital diaphragm [59,65].

### 6.3. Testicular Complications

Testicular trauma can be associated with pelvic fractures, especially in the case of motor vehicle crashes and falls from a height [66].

**Figure 14:**
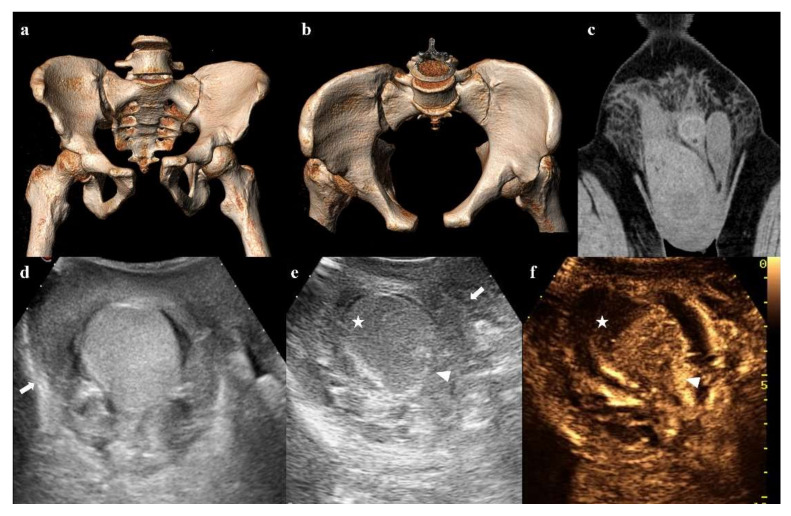
Three-dimensional volume-rendering CT reconstructions in AP and inlet views show an anterior–posterior compression fracture (**a**,**b**). Coronal CT image shows hematoma in the right inguinoscrotal region (**c**). Ultrasound shows an inhomogeneous hematoma on the extra-albuginea side (arrow in (**d**,**e**)) and a hypoechoic area in the upper part of the testis (star in (**e**)). Contrast-enhanced ultrasonography (CEUS) image clearly shows that the hypoechoic area is avascularized, as a hematoma (star in (**f**)). Ultrasound and CEUS also show the irregularity of the testicular contour (arrowhead in (**e**,**f**)). These findings are suggestive of testicular rupture.

Ultrasound and contrast-enhanced ultrasonography (CEUS) are indispensable in the proper management of these injuries. Findings of testicular trauma are hematocele, intratesticular hematoma, and testicular rupture (Figure 14) [67,68].

Magnetic resonance (MR) has great value in the evaluation of scrotal lesions, but it is rarely available in an emergency department [67,69]. Its use may be crucial in the identification of testicular rupture when ultrasound evaluation is inconclusive [70].

### 6.4. Nervous Complications

Neurological injuries are a potential complication of pelvic trauma, particularly when the sacrum is involved [71].

Fractures that involve the spinal canal or the foramina can be associated with radiculopathy, plexus dysfunction, or cauda equina syndrome [72]. Consequently, the clinical presentation is heterogeneous: motor deficit in the foot, sensor deficit, bladder incontinence, fecal incontinence, and sexual dysfunction.

Neurological complications of pelvic fractures are often overlooked in the early management of trauma [73]. MR is the best technique in the evaluation of injuries of lumbar and sacral roots and nerves [74].

## 7. Conclusions

Pelvic fractures are widely considered to be one of the most complex and fatal lesions in high-energy blunt trauma. To properly guide the patient’s management, it is crucial to understand and apply the Young and Burgess classification, which combines the mechanism and the stability of the fractures. It is also important to look for intrapelvic vascular and visceral complications, such as active bleeding, genitourinary injuries, neurological lesions, or testicular injuries.

## Figures and Tables

**Table 1:** Young and Burgess classification, and its correlations with associated intrapelvic complications and with management.

Type of Fracture	Fracture Stability	Associated Intrapelvic Complications	Management of Polytrauma Patient
*Lateral compression*		Bladder injuries Sacral nerves injuries	
Grade 1	Stable		Nonoperative management
Grade 2	Rotational instability		Surgical fixation
Grade 3	Multidirectional instability		Surgical fixation
*Anterior–posterior compression*		Vascular injuries Bladder and urethral injuries Testicular injuries	
Grade 1	Stable		Nonoperative management
Grade 2	Rotational instability		Surgical fixation
Grade 3	Multidirectional instability		Surgical fixation
*Vertical shear*	Multidirectional instability	Vascular injuries Bladder and urethral injuries Sacral nerves injuries	Surgical fixation
*Combined mechanism*	Multidirectional instability	Vascular injuries Bladder and urethral injuries Testicular injuries Sacral nerves injuries	Surgical fixation

## Data Availability

Not applicable.
